# Supplementary material for: Optimal minimal residual disease threshold in pediatric acute myeloid leukemia: A retrospective cohort study based on the TARGET database
Source: PLoS Med. 2026 May 8;23(5):e1005088. doi: 10.1371/journal.pmed.1005088 (PMC13155632; doi:10.1371/journal.pmed.1005088)
Supplement: S1 Code — (ZIP) [file pmed.1005088.s002.zip › S2 code/PROJ8_20_tbl1/PROJ8_20_tbl1.htm]

## ÑÐ¾¿ÈËÈºÃèÊö

|  |  |
| --- | --- |
|  |  |
|  | N (%) |
| MRD % at end of course 1 ·Ö×é |  |
| <0.05 | 906 (75.2%) |
| >=0.05 | 299 (24.8%) |

±íÖÐ½á¹û:
Mean(SD) Median (Min-Max) / N(%)
´Ë±íÓÃÒ×õÍ³¼ÆÈí¼þ (www.empowerstats.com) ºÍRÈí¼þÉú³É£¬Éú³ÉÈÕÆÚ£º 2026-03-18
